# A new L-shaped rigid beam FBG acceleration sensor

**DOI:** 10.1038/s41598-022-15940-x

**Published:** 2022-07-22

**Authors:** Yuntian Teng, Yewei Wang, Yixiang Tang, Xiaomei Wang, Caihua Li, Zhongchao Qiu

**Affiliations:** 1 Institute of Geophysics, China Earthquake Administration, Beijing, 100081 China; 2 Hebei Key Laboratory of Seismic Disaster Instrument and Monitoring Technology, Sanhe, 065201 China; 3 Institute of Disaster Prevention, Sanhe, 065201 China

**Keywords:** Mechanical engineering, Natural hazards, Applied optics

## Abstract

Acceleration detection is an important technology in the fields of seismic monitoring, structural health monitoring and resource exploration. A FBG acceleration sensor with the combination of L-shaped rigid beam and spring structure based on bearings is proposed against the low sensitivity that predominates in the low-frequency vibration measurement by FBG acceleration sensors, where L-shaped rigid beam is utilized to amplify the vibration signal, and is fixed by the bearings at both ends to effectively suppress the transverse crosstalk. The effects of structural parameters on the sensitivity and natural frequency of the sensors were analyzed using Origin theory, and such parameters were optimized; next, static stress and modal simulation analysis was made using COMSOL; in the end, a test system was built to test the performance of the real sensors. According to the findings, the acceleration sensor, whose natural frequency is 57 Hz, is of a flat sensitivity response in the low frequency range of 1–35 Hz, with the dynamic range being 89.83 dB, the acceleration sensitivity being up to 1241.85 pm/g, the coefficient of determination R^2^ for the sensitivity fit is 0.9997, and the transverse crosstalk being -26.20 dB within the operating frequency band. The findings offer a reference for improving the low-frequency vibration measurement capability of FBG acceleration sensors.

## Introduction

The vibration sensor based on FBG sensing technology features high sensitivity, low transmission loss, strong electromagnetic immunity, etc.^1,2^. Multiplexing FBG sensors such as acceleration, temperature, displacement, pressure, pH, humidity and magnetic field in the same optical fiber can overcome the need to use multiple sensors and heavy cables in traditional electrical sensors, as well as the shortcomings of weak anti-interference capability^3–5^. It plays an important role in vibration measurements for seismic monitoring, structural health monitoring, homeland security, aerospace, resource exploration, etc.^6^.

In recent years, FBG acceleration sensors have been extensively and intensively studied by researchers at home and abroad, who mainly focus on the structural design and elastic element selection of acceleration sensors^7,8^. At present, the elastic elements of FBG acceleration sensor mainly include cantilever beam, diaphragm, hinge and spring. Casas Ramos and Sandoval Romero^9^ proposed a new cantilever FBG vibration sensor with natural frequency of 227.3 Hz, operating bandwidth of 10–210 Hz, sensor resolution of 0.006 g, linearity and relative sensitivity error of 1.9% and ± 4.4%, respectively. Li et al.^10^ proposed an acceleration sensor based on FBG sensing with diaphragm structure, which can realize real-time decoupling and measurement of temperature and acceleration. In the range of 30–90 °C, the temperature sensitivity is 8.66 pm/°C, the acceleration sensitivity is 20.19 pm/g, and the operating bandwidth is 10–200 Hz. Yan and Liang^11^ proposed a new FBG accelerometer based on parallel double flexible hinges, which consists of two right-circular flexible hinges connected in parallel, with the measuring range being 30–200 Hz and the sensitivity being 54 pm/g. Linessio et al.^12^ proposed a two-dimensional FBG accelerometer based on omnidirectional flexible hinges, which has the function of temperature compensation and is used for the measurement of two-dimensional acceleration. However, these sensors have the disadvantages of high natural frequency, low sensitivity, etc., which make them difficult to accurately measure low-frequency vibration in engineering practice.

When designing the FBG acceleration sensor, the performance index of the sensor is the standard to measure the quality of the sensor. Several of these indicators are common, and all kinds of FBG sensors need to be used, while other indicators are hardly used. These indicators Mainly to meet the needs of special FBG sensors. Common indicators mainly include sensitivity, operating bandwidth, linearity, and lateral anti-interference ability. Aiming at the low sensitivity of FBG acceleration sensor in measuring low-frequency vibration, this paper proposes a FBG acceleration sensor with the combination of L-shaped rigid beam and spring structure based on bearings, this makes it suitable for the measurement of low-frequency vibration signals such as earthquakes and dams. Origin and COMSOL software were used to carry out theoretical and simulation analysis on the sensor structure, the real sensors were developed and a test system was built to test the sensor performance.

## Structural design and theoretical analysis of the sensor

### Structural modeling of the sensor

**Figure 1:**
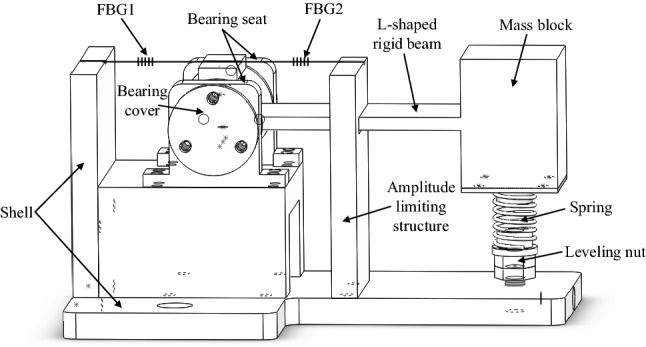
Structural modeling of the sensor.

FBG acceleration sensor with the combination of L-shaped rigid beam and spring structure based on bearings is modeled as shown in Fig. 1. It is mainly composed of shell, bearing seat (with bearings installed inside), L-shaped rigid beam, mass block, spring, leveling nut, amplitude limiting structure, etc. The bearings were installed in the bearing seat and completely fixed by two bearing covers. The mass block and the L-shaped rigid beam were kept horizontal by the leveling nut. Slots with a diameter of 1 mm were formed at the corresponding positions of the short L-shaped rigid beam, shell and amplitude limiting structure, through which two optical fibers passed and were fixed after prestressing, to avoid FBG chirp caused by attachment in grating zone. The end face of L-shaped beam was designed to be rectangular, with its width much larger than its thickness, and was fixed by the bearings at both ends, which can reduce the impact of transverse crosstalk by improving bending stiffness. The amplitude limiting structure can limit the vibration amplitude of the mass block to effectively protect FBG from being broken. By using rotating shaft, the sensor structure can be kept more stable and the impact of geometric nonlinearity can be eliminated.

When external vibration occurs, the sensor as a whole will move with the vibration signal^13^, and its acceleration will produce opposite inertial force on the mass block which will then vibrate up and down with inertial force, to drive the L-shaped beam to rotate around the bearing, and transform the vibration of the mass block into uniform strain of grating, thus affecting the center wavelength of its reflected light. The drift of the reflected light center wavelength is proportional to the linear displacement of the mass block and also proportional to the acceleration. Therefore, the acceleration can be obtained by measuring the drift of the reflected light center wavelength, so as to realize the sensing of external vibration. In addition, since two FBGs are attached to both sides of the L-shaped rigid beam respectively, the two optical fibers are deformed in opposite directions when the sensor vibrates, that is, the drift of their reflected light center wavelength is equal in magnitude and opposite in direction, and the effect of temperature on the two fibers is the same. By differentiating the two, the effects of temperature compensation and sensitivity increase can be achieved.

In the traditional cantilever beam structure, the vibration signal causes the mass block to move up and down, and the cantilever beam is placed horizontally, which is opposite to the vibration direction of the mass block, so the axial strain of the cantilever beam will be significantly reduced. In this paper, the spring is used to replace the cantilever beam structure. The displacement of the spring is consistent with the displacement of the mass block, which can better transmit the strain information and increase the sensitivity of the sensor. At the same time, the L-shaped rigid beam structure is used to amplify the displacement at the FBG by using the triangle similarity principle. , which can further increase the sensitivity of the sensor.

### Theoretical analysis

**Figure 2:**
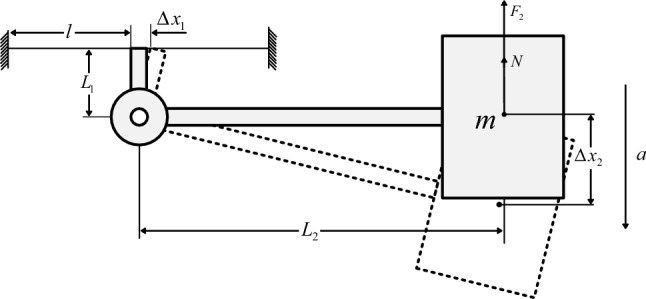
Structural vibration model.

The structural vibration model of the sensor is shown in Fig. 2. When the acceleration $a$ of vibration excitation signal acts on the sensitive direction of the sensor, the mass block will vibrate slightly around the bearing under the inertial force.

When acceleration $a$ is applied to the sensor, the displacement $\Delta x_{2}$ of the mass block relative to the shell can be expressed as

1 $$ \Delta x_{2} = \frac{a}{{w_{0}^{2} }} = \frac{ma}{K} $$

where $w_{0}$ is the natural angular frequency of the system,$m$ is the mass of the mass block,$K$ is the total elasticity coefficient (N/m) of the system composed of optical fiber and spring.

Let one of the two optical fibers extend for $\Delta x_{1}$, and the other one compress for $\Delta x_{1}$, the resultant force of the optical fibers is as follows

2 $$ F_{1} = 2k_{1} \Delta x_{1} $$

where $k_{1}$ is the elastic coefficient of optical fiber. Assumed that the Young's modulus of optical fiber is $E_{f}$, the cross-sectional area is $A_{f}$, and the effective length of optical fiber is $l$, then $k_{1} = \frac{{E_{f} A_{f} }}{l}$.

Assumed that the distance from the center of optical fiber to the center of rotating shaft is $L_{1}$, the distance from the center of mass block to the center of bearing is $L_{2}$, and the force transmitted by optical fiber to mass block is $N$, the following can be obtained by moment balance

3 $$ F_{1} L_{1} = NL_{2} $$

The force-summing analysis of the mass block shows that the elastic force produced by the deformation of the spring is $F_{2}$, and the deformation quantity is $\Delta x_{2}$, so the resultant force is $F = F_{2} + N$. The following can be obtained

4 $$ K\Delta x_{2} = k_{2} \Delta x_{2} + 2\frac{{L_{1} }}{{L_{2} }}k_{1} \Delta x_{1} $$

where $k_{2}$ is the elastic coefficient of the spring. Assumed that the shear modulus of the spring is $G$, the outer diameter is $D$, the wire diameter is $d$, and the effective number of turns of the spring is $N_{c}$, then $k_{2} = \frac{{G \times d^{4} }}{{8N_{c} \times (D - d)^{3} }}$. The following can be obtained by similar $\frac{{L_{1} }}{{L_{2} }} = \frac{{\Delta x_{1} }}{{\Delta x_{2} }}$

5 $$ K = k_{2} + 2\left( {\frac{{L_{1} }}{{L_{2} }}} \right)^{2} k_{1} $$

Then the strain $\varepsilon$ of the optical fiber is

6 $$ \varepsilon = \frac{{\Delta x_{1} }}{l} = \frac{{(L_{1} /L_{2} )ma}}{{lk_{2} + 2(L_{1} /L_{2} )^{2} E_{f} A_{f} }} $$

Finally, the sensitivity of FBG acceleration sensor is obtained as $S$

7 $$ S = 2 \cdot \lambda_{B} (1 - P_{e} )\frac{{(L_{1} /L_{2} )m}}{{lk_{2} + 2(L_{1} /L_{2} )^{2} E_{f} A_{f} }} $$

where $\lambda_{B}$ is the center wavelength of grating and $P_{e}$ is the effective elastic modulus of optical fiber.

The natural frequency $f$ of the FBG acceleration sensor is

8 $$ f = \frac{{w_{0} }}{2\pi } = \frac{1}{2\pi }\sqrt{\frac{K}{m}}  = \frac{1}{2\pi }\sqrt {\frac{{lk_{2} + 2(L_{1} /L_{2} )^{2} E_{f} A_{f} }}{lm}} $$

## Simulation analysis

### Structural parameter analysis

In the design of FBG acceleration sensors, some initial specifications of parameters related to size are needed, and the remaining parameters will be optimized to maximize the sensitivity. Since the characteristic frequency of most civil infrastructure is within 0–20 Hz, and the regional and local earthquake frequencies in natural earthquakes generally do not exceed 30 Hz, the designed acceleration sensor should exhibit a natural frequency higher than this value, but should Low enough to maximize its sensitivity and minimize noise, and should have a flat sensitivity response from 0–30 Hz.

It can be seen from Eqs. (7) and (8) that the sensitivity and natural frequency of the sensor are directly affected by the mass $m$ of the mass block, the ratio $L_{1} /L_{2}$ of short beam to long beam of L-shaped rigid beam, the spring coefficient of elasticity $k_{2}$ and the effective length $l$ of the optical fiber. The grating zone length of FBG is generally 10 mm, and the effective length of optical fiber selected for this structure is 15 mm. Origin software was used to analyze the effects of mass $m$ of the mass block, ratio $L_{1} /L_{2}$ of short beam to long beam of the L-shaped rigid beam and spring coefficient of elasticity $k_{2}$ on sensitivity and natural frequency of the sensor. The L-shaped rigid beam and mass block in the sensor were made of brass alloy with good rigidity and high density, and the spring was made of spring steel with good elasticity. The material parameters of FBG acceleration sensor are shown in Table 1.

**Table 1:** Material parameters of the sensor.

Part name	Material name	Category	Elastic modulus (Pa)	Poisson's ratio	Density (kg/m^3^)
Shell	Steel alloy	Structural Steel	2.0 × 10^11^	0.3	7850
Mass block	Brass alloy	H62	1.0 × 10^11^	0.33	8500
L-shaped rigid beam	Brass alloy	H62	1.0 × 10^11^	0.33	8500
Spring	Spring steel	65Mn	2.1 × 10^11^	0.28	7810
Bearing	Bearing steel	9Cr18	2.0 × 10^11^	0.3	7900
FBG	Glass fiber	Fiber	7.3 × 10^10^	0.17	2200

**Figure 3:**
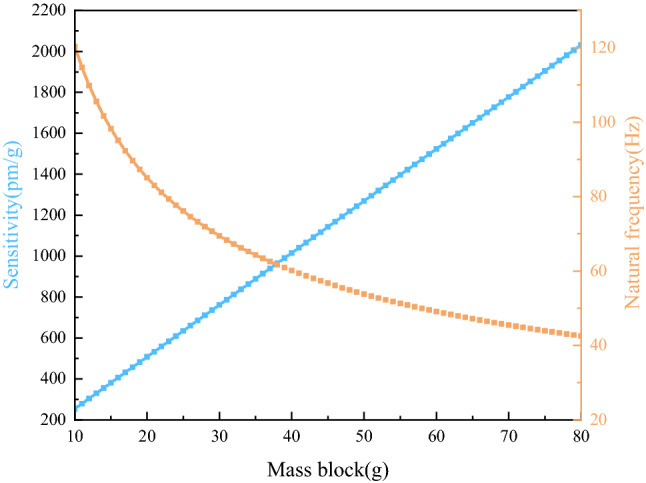
Effect of $m$ on $S$ and $f$.

Figure 3 describes the effect of the mass $m$ on the sensitivity and natural frequency of the sensor by changing the coefficient from 10 to 80 g.

As shown in Fig. 3, mass blocks with different masses had great effect on the sensitivity and natural frequency of the sensor. The larger the mass was, the higher the sensitivity and the lower the natural frequency of the sensor were. When $m$ = 10 g, the natural frequency of the sensor reached around 120 Hz and the sensitivity was only 220 pm/g; When $m$ = 80 g, the natural frequency of the sensor decreased to around 42 Hz, and the sensitivity increased to 2000 pm/g. In order to meet the requirements of low-frequency measurement, the mass of the mass block was selected as 40 g, in which case the natural frequency of the sensor was around 60 Hz and the sensitivity was over 1000 pm/g.

**Figure 4:**
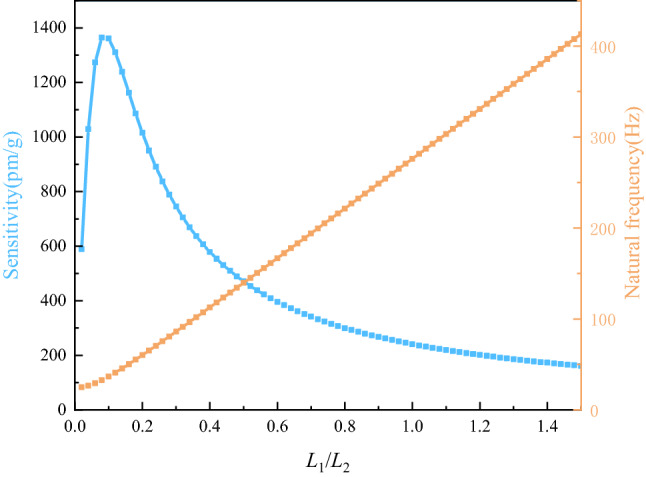
Effect of $L_{1} /L_{2}$ on $S$ and $f$.

Figure 4 describes the effect of the ratio $L_{1} /L_{2}$ on the sensitivity and natural frequency of the sensor by changing the coefficient from 0 to 1.5.

As shown in Fig. 4, with the increase of $L_{1} /L_{2}$, the natural frequency of the sensor increased continuously, and the sensitivity first increased and then decreased. When the ratio reached 0.1, the natural frequency was less than 40 Hz and the sensitivity increased to 1380 pm/g; when the ratio reached 0.2, the natural frequency reached around 60 Hz and the sensitivity was around 1100 pm/g; when the ratio reached 0.4, the natural frequency increased above 100 Hz, and the sensitivity decreased to only 250 pm/g; when the ratio was greater than 0.4, the design requirements of this paper would not be met. In order to meet the requirements of low-frequency measurement,$L_{1} /L_{2}$ was selected as 0.2.

**Figure 5:**
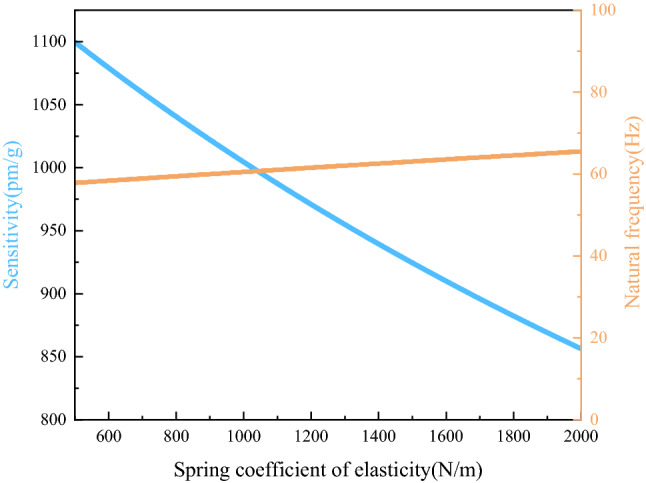
Effect of $k_{2}$ on $S$ and $f$.

Changed the spring coefficient of elasticity $k_{2}$ from 500 N/m to 2000 N/m, and substituted other parameters into the theoretical derivation formula respectively, to get the effect of $k_{2}$ on the sensitivity and natural frequency of the sensor, as shown in Fig. 5.

Figure 5 describes the effect of the spring coefficient of elasticity $k_{2}$ on the sensitivity and natural frequency of the sensor by changing the coefficient from 500 N/m to 2000 N/m.

As shown in Fig. 5, the spring coefficient of elasticity $k_{2}$ had a significant effect on the sensitivity of the sensor, but had little effect on the natural frequency. When $k_{2}$ changed from 500 N/m to 2000 N/m, the sensitivity decreased from 1100 pm/g to 860 pm/g. Therefore, the spring with elasticity coefficient of around 1000 N/m was selected as the elastic element, in which case the natural frequency of the sensor was around 60 Hz and the sensitivity was above 1000 pm/g.

### Simulation analysis by COMSOL

According to the engineering application needs of low-frequency measurement, it should be ensured that the natural frequency is higher than 50 Hz, and the sensitivity is around 1000 pm/g, taking into account the size and weight of the sensor. Based on the analysis of structural parameters, the realized structural parameters of the sensor are shown in Table 2. The sensor was modeled with Solidworks, and the results were imported into the COMSOL software for static stress and modal simulation analysis of the sensor structure.

**Table 2:** Structural parameters of the sensor.

Description	Value
Effective fiber length $l$	15 mm
Short beam of L-shaped rigid beam $L_{1}$	9 mm
Long beam of L-shaped rigid beam $L_{2}$	45 mm
Mass of mass block $m$	40 g
Bearing	2 × 6 × 2.3 mm
Spring	0.6 × 5.8 × 9.5 mm
Center wavelength of FBG1	1565.5 nm
Center wavelength of FBG2	1555.5 nm
Cross-sectional area of fiber $A_{f}$	1.23 × 10^–8^ m^2^
Effective elasticity coefficient of optical fiber $P_{e}$	0.22

**Figure 6:**
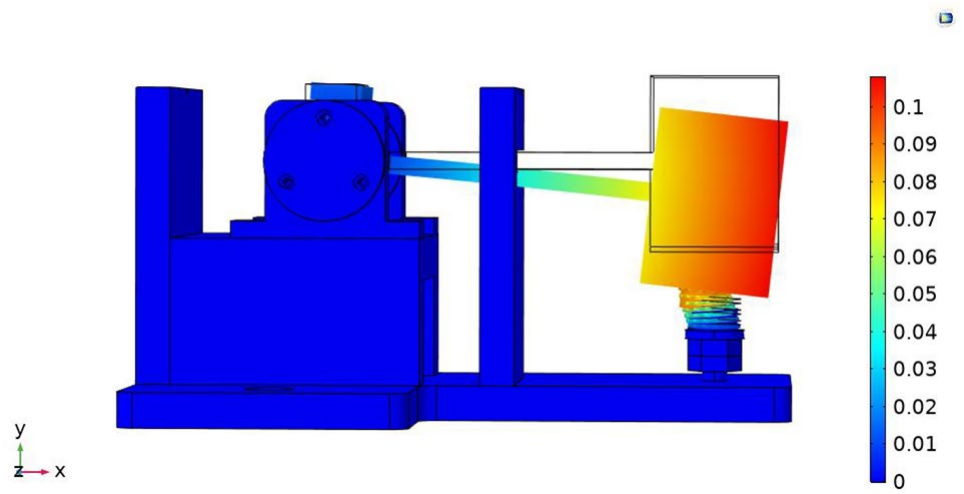
Static stress analysis of structure.

A fixed constraint was applied to the bottom end of the sensor, and an acceleration of 2 g was applied on the whole to obtain the equivalent displacement diagram of the model, as shown in Fig. 6. It could be known that the displacement of deformation at the free end was the largest, which decreased gradually from the free end to the supporting end, the maximum displacement of mass block was 0.108 mm, the displacement of rigid beam at the amplitude-limiting position was 0.033 mm, and the displacement of L-shaped rigid beam at the position where optical fiber was attached was 0.014 mm. Therefore, the sensor structure can respond to the displacement and strain at the free end, and the deformation does not affect the physical properties of optical fiber, which can ensure the stability of the sensor.

**Figure 7:**
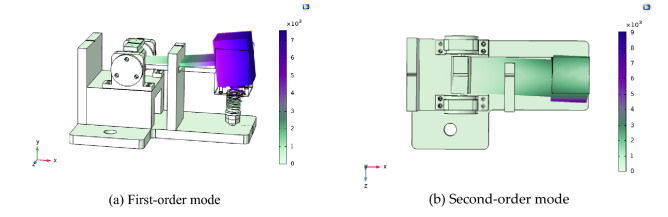
Modal analysis of structure.

The model was put into the modal analysis module, and was meshed as a whole. The first four-order modes of the model were analyzed, and the frequencies of the first-order, second-order, third-order, and fourth-order modes identified were 60.71 Hz, 457.53 Hz, 694.01 Hz and 930.66 Hz respectively. That is to say, the natural frequency of the structure was 60.71 Hz, which achieves the goal that the natural frequency of the sensor after the optimization of the structure parameters is about 60 Hz. And the difference between the frequency of first-order mode and the frequencies of second-, third-, and fourth-order modes was large, indicating that its cross coupling was small and the cross interference was effectively reduced. First-order, and second-order are shown in Fig. 7.

## Experimental test and analysis of the sensor

### Sensor development and test system building

**Figure 8:**
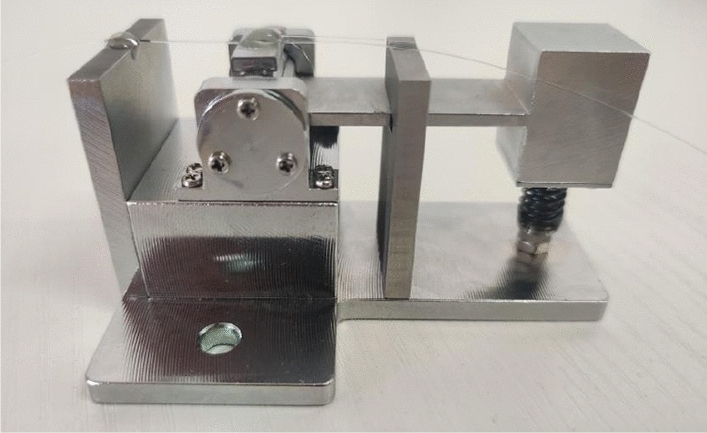
Physical prototype of FBG acceleration sensor.

According to the results of theoretical and simulation analysis, the developed FBG acceleration sensor is shown in Fig. 8. Before the test, optical fibers were attached by the two-point attachment technique, to avoid chirp caused by attachment in grating zone. At first, one end of the optical fiber was placed in the slot of the short beam of L-shaped rigid beam, adhered by UV glue, and fixed after irradiation by UV lamp for 40 s. The end of the optical fiber was prestressed by a weight of 20 g, and then fixed by UV glue and UV lamp. The second optical fiber was attached in the same way as the first one.

**Figure 9:**
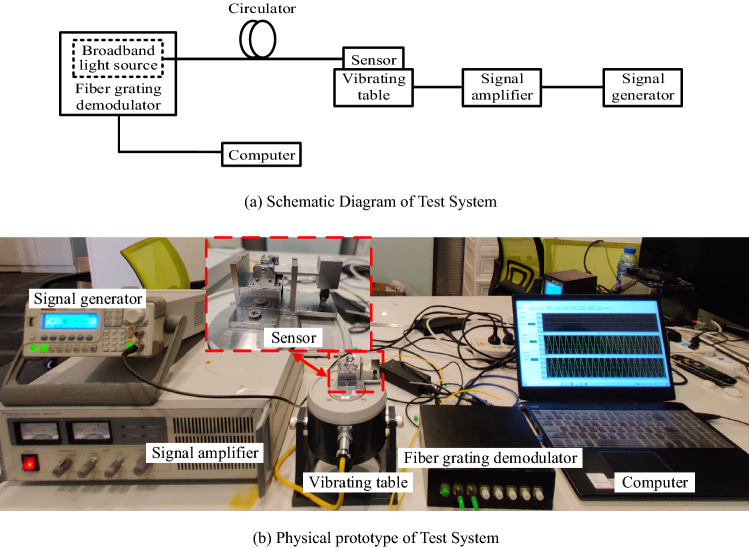
Test system of the sensor.

The experimental vibration system consists of FBG acceleration sensor, vibrating table, signal generator, signal amplifier, fiber grating demodulator (built-in light source) and computer, as shown in Fig. 9. The vibrating table system includes a MWY-JZQ50 calibrating table, with a maximum amplitude of 12.5 mm and a maximum acceleration of 45.5 g; signal function generator with sampling rate of 1 GSa/a, which has 14 quasi-waveform functions and abundant standard configuration interfaces; and signal amplifier (MWY-TZQ50), whose frequency response range is 1–15,000 Hz, and signal-to-noise ratio is greater than 75 dB, matched with signal function generator to amplify function signals. The fiber grating demodulator (MWY-FBG-CS800) used in the experiment can demodulate wavelengths in the range of 1528–1568 nm, with a maximum sampling rate of 1 kHz and a resolution of 0.1 pm. The circulator, which is used as a connecting part, transmits the light wave of broadband light source to the acceleration sensor. After passing through the FBG, the light in a certain wavelength range is reflected back, and then transmitted to the demodulator through the circulator to demodulate the information carried by the wavelength change of light wave.

### Temperature response test

**Figure 10:**
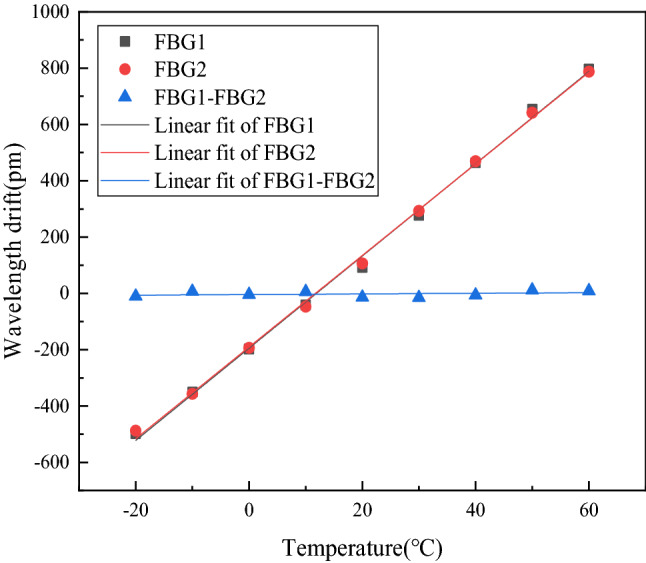
Effect of temperature on center wavelength.

The experimental device for temperature response test consists of FBG acceleration sensor, temperature control box, fiber grating demodulator and computer. The environmental temperature was set as the only variable in the experiment; FBG acceleration sensor was put into the temperature control box, which could ensure other environmental parameters unchanged to effectively achieve the purpose of controlling variables. Set the initial temperature change point of the temperature control box as −20 °C, the end point as 60 °C, and the step size as 10 °C. When the temperature of each node reached equilibrium, kept it for 2 min, then measured the variation of FBG center wavelength, recorded and analyzed the data, and finally normalized the data, as shown in Fig. 10.

As shown in Fig. 10, the change of temperature would seriously affect the measurement precision and accuracy of FBG acceleration sensor. The effect of temperature change on a single FBG was 16.32 pm/°C, and when the measured ambient temperature changed up to 60 °C, the effect on the center wavelength of FBG reached 800 pm, so it is very important to eliminate the effect of temperature in vibration monitoring. However, the effect of temperature on the double-FBG acceleration sensor was 0.11 pm/°C, which indicates that the double-fiber structure can significantly improve the effect of the measured ambient temperature on the FBG acceleration sensor, and can realize the function of self-compensation for the change of ambient temperature.

### Output response characteristics

**Figure 11:**
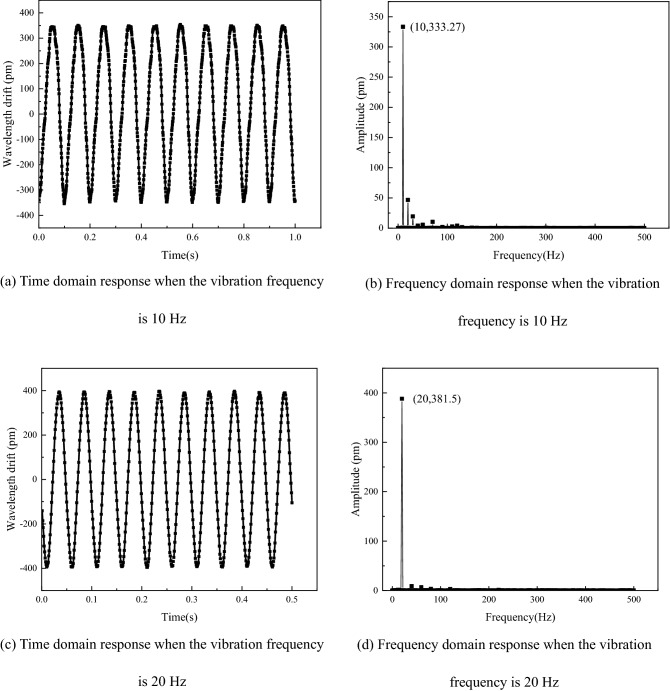
Output response curve of the sensor.

To test the response characteristics of the sensor, the output acceleration amplitude of the vibrating table was set as 3 m/s^2^, and the vibration frequencies were 10 Hz and 20 Hz, respectively. The time-domain signals measured by the FBG acceleration sensor and the corresponding frequency domain signals are shown in Fig. 11.

As shown in Fig. 11, the measured signal was of good quality, and the sensor could well obtain sinusoidal excitation input from outside. The intact sine wave showed that FBG was under uniform stress, without chirp and multi-peaks. The amplitudes of time domain curves measured at two different frequency points under the same acceleration input were similar, which proves that the sensor has a flat response in the operating frequency band.

### Amplitude frequency characteristics and dynamic range test

**Figure 12:**
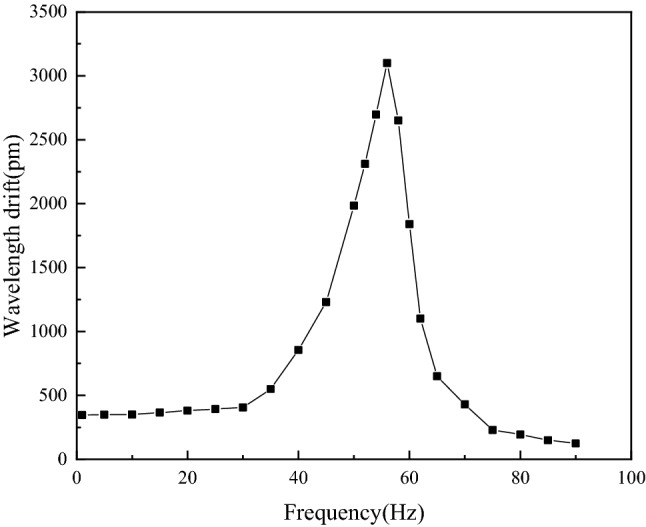
Amplitude-frequency characteristics of the sensor.

The acceleration of the vibrating table was set as 3 m/s^2^, 1–90 Hz excitation was generated by signal generator, the output frequency of the vibrating table was changed at a step of 5 Hz which was deceased to 2 Hz when approaching the natural frequency of the sensor, and the variations in center wavelengths of FBG1 and FBG2 were recorded. The amplitude-frequency response curve of acceleration sensor was obtained by calculation, as shown in Fig. 12.

As shown in Fig. 12, the sensor had a good flat zone at 1–35 Hz, and 1 Hz was the minimum vibration frequency that can be generated by the signal generator. The natural frequency of the sensor was 57 Hz, which is close to the theoretical value of 60.71 Hz in the simulation analysis, the error might result from the losses of the sensor during processing and the prestress applied when the optical fiber was attached.

Dynamic range is one of the important parameters of the sensor. For FBG acceleration sensor, the dynamic range can be expressed as the logarithmic ratio of the maximum drift $\lambda_{\max }$ of FBG center wavelength to the resolution $\lambda_{\min }$ of demodulation system, and its relational expression is as follows

9 $$ D_{R} = 20\lg (\frac{{\lambda_{\max } }}{{\lambda_{\min } }}) $$

In the test, the maximum drift of FBG center wavelength was 3100 pm, and the resolution of fiber grating demodulator used in the test system was 0.1 pm. The dynamic range of FBG acceleration sensor was calculated as 89.83 dB.

### Sensitivity and linear response test

**Figure 13:**
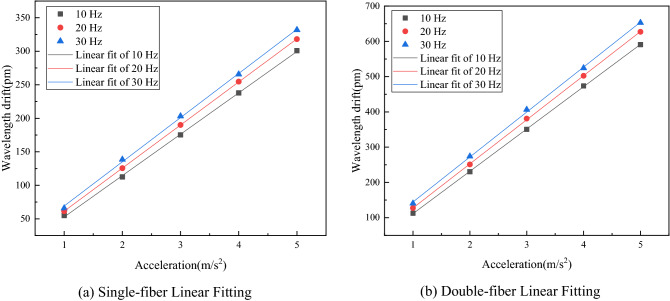
Sensor linear fitting curve.

The sensitivity of the sensor determines the ability of FBG acceleration sensor to pick up weak vibration signals. Sensitivity of FBG acceleration sensor is the ratio of FBG output wavelength variation to input acceleration at a certain operating frequency. During the test of FBG acceleration sensor sensitivity, it is necessary to determine not only the ratio of FBG output wavelength variation to input acceleration, but also the linearity of the ratio of FBG output wavelength variation to input acceleration. The better the linear response is, the more stable the operating performance of the sensor is. To determine the sensitivity of the sensor, set the output frequencies of the vibrating table as 10 Hz, 20 Hz and 30 Hz respectively, changed the acceleration at a step of 1 m/s^2^, with the variation range of 1–5 m/s^2^, and recorded the variations of the center wavelengths of FBG1 and FBG2 at two different frequencies. The plotted linear fit line is shown in Fig. 13.

According to the analysis of collected data, when the input frequency was 10 Hz, 20 Hz and 30 Hz, the single-fiber sensitivity of the acceleration sensor was 617.5 pm/g, 642.9 pm/g and 659.4 pm/g respectively, the double-fiber sensitivity was 1199.75 pm/g, 1250.9 pm/g and 1274.9 pm/g, respectively, which achieves the goal of the sensor sensitivity above 1000 pm/g after structural parameter optimization. And the fit coefficient of determination R^2^ of double fiber at 10 Hz, 20 Hz and 30 Hz was 0.9995, 0.9999 and 0.9997, respectively. The results show that there is a good linear relationship between the center wavelength drift of FBG acceleration sensor and the input acceleration amplitude, and double fiber can increase the sensitivity. The minimum detectable acceleration of the sensor in the test was 0.05 m/s^2^.

### Impulse response experiment

**Figure 14:**
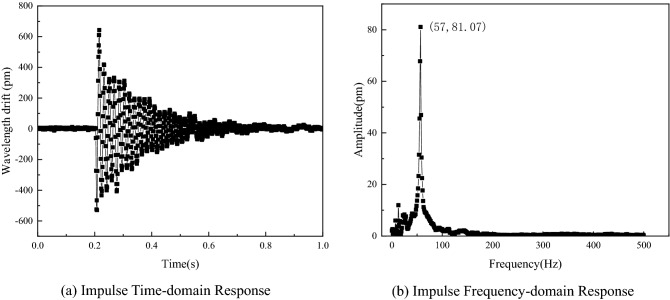
Impulse response curve.

Impulse is a transient process with high acceleration amplitude and containing more vibration frequency information, and impulse response mainly depends on the structural properties of the measured object. Therefore, an impulse response experiment was conducted to verify the natural frequency of the sensor. In the experiment, the method of instantaneously hitting the vibrating table is used to simulate the generation of a shock signal. The output of the sensor is shown in Fig. 14. Figure 14a shows the time-domain curve of impulse response, and Fig. 14b shows the frequency-domain response after FFT of time-domain signal.

From the time domain output of the sensor, it can be seen that before the shock signal is generated, the center wavelength of the FBG has zero drift and remains stable. The shock signal was generated at 0.2 s, and the maximum drift of the center wavelength was 640 pm, and then gradually weakened. At 0.7 s, the center wavelength drift returned to zero and remained stable. From the frequency domain output of the sensor, it can be seen that the peak of the impulse response amplitude is at 57 Hz, and there are no other obvious peaks in the frequency domain output. From the side, it indicates that the natural frequency of the sensor is about 57 Hz, which is consistent with the amplitude and frequency response test results, further verifying the correctness of the experimental results.

### Transverse interference immunity test

Vibration signal is a kind of vector signal with directionality, so for single-degree-of-freedom FBG acceleration sensor, its transverse interference immunity must be considered. The transverse crosstalk of the sensor is the logarithmic ratio of FBG center wavelength drift when the acceleration excitation signal acts on the cross direction and FBG center wavelength drift when the acceleration excitation signal acts on the main direction at the same frequency.

**Figure 15:**
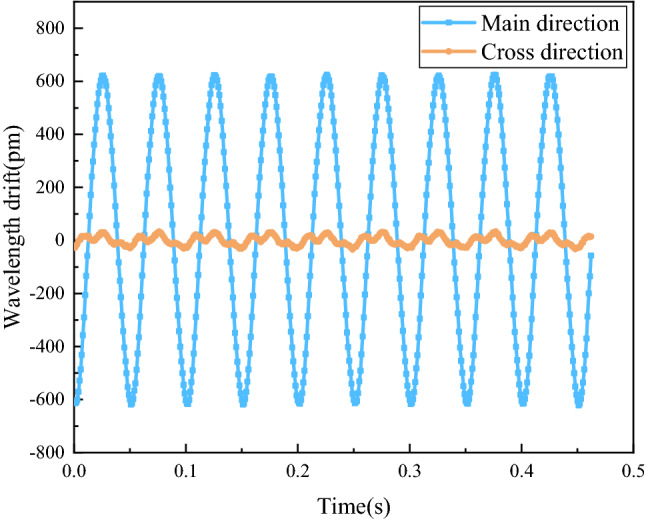
Transverse interference immunity test.

The sensor was fixed on the vibrating table, and a sinusoidal excitation signal with a frequency of 20 Hz and an acceleration of 5 m/s^2^ was set. In the same vibration environment, the drifts of FBG center wavelength under transverse vibration and longitudinal vibration of the sensor were recorded, as shown in Fig. 15. According to the results, the longitudinal response and transverse response of the sensor were 616.95 pm and 30.2 pm, respectively, so the transverse crosstalk was calculated as -26.20 dB, which indicates that the sensor can be regarded as a single-degree-of-freedom vibration under vibration conditions and has strong transverse interference immunity.

### Sensor performance analysis

Through the above experiments, the performance of the L-shaped rigid beam structure proposed in this paper is tested, and its performance comparison with the sensor introduced in the introduction is shown in Table 3.

**Table 3:** Structural parameters of the sensor.

Structure name (author)	Natural frequency	working bandwidth	Sensitivity	Lateral crosstalk	Whether temperature compensation
Cantilever-type FBG mechanical vibration sensor(Miguel A.)	227.3 Hz	10–210 Hz	391 pm/g	–	No
Diaphragm Based FBG Acceleration Sensor(Tianliang Li)	600 Hz	10–200 Hz	20.19 pm/g	−29.60 dB	Yes
FBG accelerometer based on parallel double flexible hinges(Bing Yan)	800 Hz	30–200 Hz	54 pm/g	−26.74 dB	No
Biaxial Optical Fiber Accelerometer(Linessio)	747.5 Hz	30–240 Hz	100 pm/g	–	No
L-shaped Rigid Beam FBG Acceleration Sensor(this paper)	57 Hz	1–35 Hz	1241.85 pm/g	−26.20 dB	Yes

It can be seen from Table 3 that the L-shaped rigid beam FBG acceleration sensor proposed in this paper has a lower natural frequency, which enables it to measure low-frequency vibration signals below 30 Hz such as earthquakes and dams. Compared with other structures, the structure adopts the "bearing—spring" system and L-shaped rigid beam amplify the displacement at the FBG, which can further increase the sensitivity of the sensor.

## Conclusions

A FBG acceleration sensor with the combination of L-shaped rigid beam and spring structure based on bearings is proposed against the low sensitivity that predominates in the low-frequency vibration measurement by FBG acceleration sensors, where L-shaped rigid beam is utilized to amplify the vibration signal, and is fixed by the bearings at both ends to effectively suppress the effect of transverse crosstalk, and the amplitude limiting structure can limit the vibration amplitude of the mass block to effectively protect FBG from being broken. According to the findings, the acceleration sensor has a natural frequency of 57 Hz and an acceleration sensitivity up to 1241.85 pm/g, which is close to the theoretical analysis. The errors might result from the processing and installation errors of the sensor, amount of adhesive, FBG prestress, friction existing in the rotation of bearing, accuracy of experimental vibration equipment, etc. The acceleration sensor is of a flat sensitivity response in the low frequency range of 1–35 Hz, with the dynamic range being 89.83 dB, the coefficient of determination R^2^ for the sensitivity fit is 0.9997, and the transverse crosstalk being -26.20 dB within the operating frequency band, which can realize the real-time and high-precision measurement of low-frequency weak vibration signal.

## Data Availability

The datasets used and/or analysed during the current study are available from the corresponding author on reasonable request.
